# Supplementary material for: Identifying therapeutic targets for cancer among 2074 circulating proteins and risk of nine cancers
Source: Nat Commun. 2024 Apr 29;15:3621. doi: 10.1038/s41467-024-46834-3 (PMC11059161; doi:10.1038/s41467-024-46834-3)
Supplement: Supplementary file 1 — Supplementary Info [file 41467_2024_46834_MOESM1_ESM.pdf]

Supplementary Information

Collection and quality control for cis pQTL. We gathered summary statistics from publicly available protein GWAS'[1–4](See Supplementary Table 1 and Supplementary Table 1). We subsequently extracted all independent cis-pQTL [clumping at $r^2 < 0.01$], defined as independent SNPs associated with a protein concentration in blood lying within 1 megabase of a protein's cognate gene with at least $p < 5x10^{-08}$. Further, given our strong a priori hypothesis to observe SNP associations with circulating protein concentrations at or adjacent to its cognate gene, we have re-processed previously published protein GWAS on the OpenGWAS platform to identify additional *cis* pQTL significant at $p < 5x10^{-05}$. Specifically, for each protein GWAS where full summary statistics were available[5], we extracted a genomic region 1 megabase up and downstream of a protein's cognate gene. Where no cis-pQTL was present at $p < 5x10^{-08}$, we extracted all cis-pQTL present at $p < 5x10^{-05}$. All variants identified were then clumped at $r^2 < 0.01$ using 1000 genomes CEU population to select independent cis-pQTL. We additionally excluded SNPs based on weak instrument strength at $F_{stat} < 10$ [β^2/σ^2].

Supplementary Table 1. Details on studies reporting *cis* pQTL included in cancer risk analyses

Author	Publication Year	Platform	Sample Size	Population	Notes
Zheng et al.[1]	2020	Olink/Somalogic/xMAP	Various	European	Study aggregating previous protein GWAS
Folkersen et al.[3]	2020	Olink	21,756	European	
Ferkingstad et al.[2]	2021	Somalogic	35,559	European	
Pietzner et al.[4]	2021	Somalogic	10,708	European	

Cancer GWAS summary statistics. Nine cancer outcomes and their subtypes (where applicable/available) were considered in this study, including cancer of the bladder, breast, endometrium, head and neck, lung, ovary, pancreas, kidney, and malignant non-melanoma. **Bladder.** GWAS summary statistics were obtained for bladder cancer risk [ICD-10: C67; 8,988 cases and 11,978 controls] from a study of participants with European ancestry[6]. **Breast.** GWAS Summary statistics for breast cancer risk overall [ICD-10: C50; 133,384 cases and 113,789 controls] and stratified by molecular subtypes [luminal B-like, luminal A-like, luminal /HER2-negative-like, HER2-enriched-like, and triple negative][7] and by oestrogen receptor status [ER-positive: 69,501 cases and 105,974 controls; ER-negative: 21,468 cases and 105,974 controls][8]. All analyses were in participants of European ancestry. **Endometrium**. Endometrial cancer [ICD-10: C54.1] risk GWAS summary statistics from the Endometrial Cancer Association Consortium from 121,885 participants of European ancestry [12,906 cases and 108,979 controls][9]. **Head and Neck**. GWAS summary statistics for head and neck cancer [6,034 cases and 6,585 controls] based on ICD-10 codes: oral cavity [2,641 cases] (C02.0–C02.9, C03.0–C03.9, C04.0–C04.9 and C05.0–C06.9) and oropharynx [2,990 cases] (C01.9, C02.4 and C09.0–C10.9). This study population comprised participants from Europe (45.3%), North America (43.9%) and South America (10.8%)[10]. **Lung**. GWAS summary statistics for lung cancer [ICD-10: C34] risk overall were extracted from a recent large-scale lung cancer meta-analysis between a large case-control GWAS for lung cancer [29,266 cases and 56,450 controls] and a GWAS of family history of lung cancer in the UK Biobank [48,843 proxy cases and 195,387 controls][11]. Total effective sample size was 41,477 cases and 105,297 controls. Summary statistics for histological subtype were taken from INTEGRAL-ILCCO (adenocarcinoma: 11,273 cases, 55,483 controls; squamous cell carcinoma: 7,426, 55,627; small cell carcinoma: 2,664, 21,444) and by smoking status (never: 2,355, 7,504; ever: 23,223, 16,964)[12]. All analyses were in participants of European ancestry. **Ovary**. GWAS summary statistics were obtained for invasive epithelial ovarian cancer [ICD-10: C56; 25,509 cases and 40,941 controls] among European ancestry participants in the Ovarian Cancer Association Consortium (OCAC)[13]. Data were also extracted for histological subtypes: high-grade serous carcinoma [13,037 cases], endometrioid carcinoma [2,810 cases], invasive mucinous ovarian cancer [1,417 cases], and clear cell carcinoma [1,366 cases], and high/low grade serous carcinoma [14,049 cases]. All subtype GWAS had 40,941 controls. **Pancreas**. GWAS data from pancreatic cancer cases of European ancestry [ICD-10: C25; 7,638 cases and 7,364 controls] were obtained from the PanScan (12 studies) and PanC4 (10 studies) consortia[14] through the National Center for Biotechnology Information database of Genotypes and Phenotypes

(dbGaP; Study Accession: phs000206.v3.p2 and phs000648.v1.p1; project reference #9314). **Kidney**. We obtained GWAS summary statistics for renal cell carcinoma [ICD-10: C64] in European ancestry [10, 784 cases and 20, 406 controls][15]. **Malignant non-melanoma.** GWAS summary statistics for malignant non-melanoma skin cancer in European ancestry participants from the UK Biobank [ICD-10: C43; 23,694 cases and 372,016 controls] from the OpenGWAS public database[16].

MR and colocalization analyses. Cis-pQTL were harmonised with GWAS summary statistics for each cancer outcome by matching on rsID where directly available, and by selecting a proxy SNP at r^2_{max} where $r^2 > 0.8$ in 1000 Genomes Project CEU (European-heritage) population with the index cis-pQTL where necessary. Harmonised SNPs were oriented to the protein-increasing allele. Primary risk estimates were odds ratios estimated using per-cis-pQTL Wald Ratios [$\beta_{outcome}/\beta_{protein}$]. All MR associations with $p_{Wald} < 0.05$ were subsequently evaluated for confounding by linkage disequilibrium (LD). Specifically, colocalization was assessed for 150kb window centred on the index cis-pQTL using two approaches: conventional colocalization[17,18], which tests for the presence of a single shared genetic signal and conditional iterative colocalization[18]; both methods estimate the posterior probability [PP4] of a shared causal variant using Bayes factors, however, only the conditional iterative method allows for the possibility of multiple independent (but partially correlated) causal variants in proximity[19]. LD matrices for conditional iterative analyses were calculated using the 1000 Genomes Project CEU reference population. Colocalization priors were *p1*=1E-3, *p2*=1E-4, *p12*=1E-5, which approximately equates to an 80% prior belief that there is only a signal in the protein GWAS and a 0.001% prior belief in favour of colocalization. The greatest PP4 from these two colocalization methods [$PP4_{max}$] was used and we considered $PP4_{max} > 0.7$ as indicating cis-pQTL MR associations were unlikely to have be confounded by LD. Cis-pQTL with Bonferroni significant associations (i.e., $p_{Wald} < 0.05/N_{Proteins}$ where $N_{Proteins}$ is the number of unique proteins analysed for a given cancer outcome) that also had evidence of colocalization (i.e., $PP4_{max} > 0.7$) were considered noteworthy and subjected to further follow-up analyses. Where multiple independent [$r^2 < 0.01$] cis-pQTL, proxying the same protein, had noteworthy associations with the same cancer outcome they were combined using the inverse-variance weighted approach[20].

Replication of candidate aetiological proteins for cancer risk. Where data were available, we conducted a replication of noteworthy *cis* pQTL MR associations (i.e. $PP4_{max} > 0.7$ & Bonferroni p_{Wald}) using external cancer GWAS data from either a meta-analysis of FinnGen r9[21] and the UK Biobank[22], or from FinnGen alone depending on the endpoint (see Table 1 for case counts). We additionally calculated a cis-pQTL MR Wald ratio using a combined cancer GWAS estimate for the cis-pQTL from a fixed effect meta-analysis of initial and replication cancer GWAS summary statistics. We considered directionally concordant risk estimates and cis-pQTL MR Wald ratio $p < 0.05$ in independent replication data to indicate replication. No sample overlap was present between samples used to generate protein associations and used to conduct replication analyses apart from lung cancer overall summary statistics. Lung cancer overall GWAS is a meta-analysis between data from the INTEGRAL-ILCCO consortium and a GWAS of family history for lung cancer in the UK Biobank, which excluded lung cancer cases. Therefore, modest overlap exists among controls used in Finngen+UK Biobank lung cancer GWAS.

MR and colocalization PHEWAS analyses. We conducted additional analyses to provide greater context to the specificity of a noteworthy protein association with cancer risk using PHEWAS MR and colocalization analyses as well as consulting several public databases. We performed these steps to collate information on potential harmful or additional beneficial consequences of altering identified protein concentrations in human populations. Firstly, we assessed the association of each protein with all available traits on the OpenGWAS platform[23] where cis-pQTL p < cancer-specific Bonferroni threshold established above using MR and colocalization as described above. Only traits with full information needed for analyses were included, such as sample size and case proportion. Expression traits were not included. All protein-trait associations identified with PP4 > 0.7 in PHEWAS MR and colocalization analyses were subsequently collectively analysed using HyprColoc[24] to assess the posterior probability that a cis-pQTL window had a shared causal signal across the protein of interest, traits identified in OpenGWAS, and the cancer outcome of interest. Results with HyprColoc PP4 > 0.7 are reported. Secondly, we conducted a search of several relevant databases with information on probability of loss of function intolerance (pLI), exome-sequencing studies, rare-variant association studies, and Mendelian genetics not likely to overlap with OpenGWAS traits, including Online Catalog of Human Genes and Genetic Disorders (OMIM)[25], Genebass[26], and the AstraZeneca PheWAS Portal[27]. For Genebass, we report potential loss of function (pLOF) variation associations with traits where SKAT-O p < recommended threshold of 2.5×10^{-6}, while for results in the AstraZeneca PheWAS Portal we consider gene-trait associations that pass the

recommended threshold of $p < 2.1 \times 10^{-9}$. For OMIM database, we considered reports presented for allelic variants.

Methods for the target classification, drug analysis, and clinical trial analysis. UniProt and NIH Pharos Consortium databases were used to obtain information about cancer risk proteins. Information on drugs and associated targets was obtained using the most recent release of the DrugBank and NIH Pharos Consortium. In DrugBank, drugs were mapped to their main identifiers (international non-proprietary name if available). Drug-Target interactions were considered as is, and we included interactions with pharmacological action "yes" and "unknown" and excluded agents with pharmacological action "no". Drug-Target mapping was performed through gene names and did not distinguish protein isoforms encoded by the same gene. Approval of drugs was checked using information from U.S. Food and Drug Administration (FDA) via provided Drugs@FDA search portal. Mapping to the pharos consortium was performed through a target gene name. Ligand lists were downloaded for every investigated target. We grouped ligands based on their names, and clinical trial mapping was performed for agents that had a drug-like name or a code identifier (but not SID or CHEMBL id). Clinical status of the drug was checked based on its appearance in the ClinicalTrials.gov. Drugs were considered clinical if they were detected in ClinicalTrials.gov but did not reach FDA approval. Information on associated trials was collected both overall and for a specific target-disease pairs. Clinical trials were characterized based on their associated phases. These phases were classified in the order starting from the earliest phase. The following classification was considered: Not Applicable, Early Phase 1, Phase 1, Phase 1|Phase 2, Phase 2, Phase 2|Phase 3, Phase 3, Phase 4. Some of the trials contained two phases simultaneously, and thus both phases were included. Trials were grouped based on their activity status. All statuses including: Active, not recruiting, Enrolling by invitation, Not yet recruiting, Recruiting were considered as Active, whereas all other statuses were labeled as Not active. Targets were also labeled based on their development activity status. A target was considered Active if there was any related trial with active status during the data analysis. If the last completed study was earlier than three years before the data analysis, the target development was considered Inactive. Otherwise, it was classified as Probably active.

References

1. Zheng, J. *et al.* Phenome-wide Mendelian randomization mapping the influence of the plasma proteome on complex diseases. *Nat Genet* **52**, 1122–1131 (2020).
2. Ferkingstad, E. *et al.* Large-scale integration of the plasma proteome with genetics and disease. *Nat Genet* **53**, 1712–1721 (2021).
3. Folkersen, L. *et al.* Genomic and drug target evaluation of 90 cardiovascular proteins in 30,931 individuals. *Nat Metab* **2**, 1135–1148 (2020).
4. Pietzner, M. *et al.* Mapping the proteo-genomic convergence of human diseases. *Science (1979)* **374**, (2021).
5. Sun, B. B. *et al.* Genomic atlas of the human plasma proteome. *Nature* **558**, 73–79 (2018).
6. Rothman, N. *et al.* A multi-stage genome-wide association study of bladder cancer identifies multiple susceptibility loci. *Nat Genet* **42**, 978–984 (2010).
7. Zhang, H. *et al.* Genome-wide association study identifies 32 novel breast cancer susceptibility loci from overall and subtype-specific analyses. *Nat Genet* **52**, 572–581 (2020).
8. Michailidou, K. *et al.* Association analysis identifies 65 new breast cancer risk loci. *Nature* **551**, 92–94 (2017).
9. O'Mara, T. A. *et al.* Identification of nine new susceptibility loci for endometrial cancer. *Nat Commun* **9**, 3166 (2018).
10. Lesseur, C. *et al.* Genome-wide association meta-analysis identifies pleiotropic risk loci for aerodigestive squamous cell cancers. *PLoS Genet* **17**, e1009254 (2021).
11. Gabriel, A. A. G. *et al.* Genetic Analysis of Lung Cancer and the Germline Impact on Somatic Mutation Burden. *JNCI: Journal of the National Cancer Institute* **114**, 1159–1166 (2022).
12. McKay, J. D. *et al.* Large-scale association analysis identifies new lung cancer susceptibility loci and heterogeneity in genetic susceptibility across histological subtypes. *Nat Genet* **49**, 1126–1132 (2017).
13. Phelan, C. M. *et al.* Identification of 12 new susceptibility loci for different histotypes of epithelial ovarian cancer. *Nat Genet* **49**, 680–691 (2017).

14. Klein, A. P. *et al.* Genome-wide meta-analysis identifies five new susceptibility loci for pancreatic cancer. *Nat Commun* **9**, 556 (2018).
15. Scelo, G. *et al.* Genome-wide association study identifies multiple risk loci for renal cell carcinoma. *Nat Commun* **8**, 15724 (2017).
16. Kimberley Burrows & Philip Haycock. Genome-wide Association Study of Cancer Risk in UK Biobank. *10.5523/bris.aed0u12w0ede20olb0m77p4b9*.
17. Giambartolomei, C. *et al.* Bayesian Test for Colocalisation between Pairs of Genetic Association Studies Using Summary Statistics. *PLoS Genet* **10**, e1004383 (2014).
18. Wallace, C. A more accurate method for colocalisation analysis allowing for multiple causal variants. *PLoS Genet* **17**, e1009440 (2021).
19. Deng, Y. & Pan, W. A powerful and versatile colocalization test. *PLoS Comput Biol* **16**, e1007778 (2020).
20. Burgess, S., Butterworth, A. & Thompson, S. G. Mendelian Randomization Analysis With Multiple Genetic Variants Using Summarized Data. *Genet Epidemiol* **37**, 658–665 (2013).
21. Kurki, M. I. *et al.* FinnGen provides genetic insights from a well-phenotyped isolated population. *Nature* **613**, 508–518 (2023).
22. Hewitt, J., Walters, M., Padmanabhan, S. & Dawson, J. Cohort profile of the UK Biobank: diagnosis and characteristics of cerebrovascular disease. *BMJ Open* **6**, e009161 (2016).
23. Hemani, G. *et al.* The MR-Base platform supports systematic causal inference across the human phenome. *Elife* **7**, (2018).
24. Foley, C. N. *et al.* A fast and efficient colocalization algorithm for identifying shared genetic risk factors across multiple traits. *Nat Commun* **12**, 764 (2021).
25. Amberger, J., Bocchini, C. A., Scott, A. F. & Hamosh, A. McKusick's Online Mendelian Inheritance in Man (OMIM(R)). *Nucleic Acids Res* **37**, D793–D796 (2009).
26. Karczewski, K. J. *et al.* Systematic single-variant and gene-based association testing of thousands of phenotypes in 394,841 UK Biobank exomes. *Cell Genomics* **2**, 100168 (2022).
27. Wang, Q. *et al.* Rare variant contribution to human disease in 281,104 UK Biobank exomes. *Nature* **597**, 527–532 (2021).
