# Supplementary material for: Identifying therapeutic targets for cancer among 2074 circulating proteins and risk of nine cancers
Source: Nat Commun. 2024 Apr 29;15:3621. doi: 10.1038/s41467-024-46834-3 (PMC11059161; doi:10.1038/s41467-024-46834-3)
Supplement: Supplementary file 3 — Reporting Summary [file 41467_2024_46834_MOESM3_ESM.pdf]

nature portfolio

Corresponding author(s): Karl Smith-Byrne

Last updated by author(s): 2023-5-18

Reporting Summary

Nature Portfolio wishes to improve the reproducibility of the work that we publish. This form provides structure for consistency and transparency in reporting. For further information on Nature Portfolio policies, see our Editorial Policies and the Editorial Policy Checklist.

Statistics

For all statistical analyses, confirm that the following items are present in the figure legend, table legend, main text, or Methods section.

n/a	Confirmed	
[ ]	[x]	The exact sample size (*n*) for each experimental group/condition, given as a discrete number and unit of measurement
[ ]	[x]	A statement on whether measurements were taken from distinct samples or whether the same sample was measured repeatedly
[ ]	[x]	The statistical test(s) used AND whether they are one- or two-sided<br>*Only common tests should be described solely by name; describe more complex techniques in the Methods section.*
[x]	[ ]	A description of all covariates tested
[ ]	[x]	A description of any assumptions or corrections, such as tests of normality and adjustment for multiple comparisons
[ ]	[x]	A full description of the statistical parameters including central tendency (e.g. means) or other basic estimates (e.g. regression coefficient) AND variation (e.g. standard deviation) or associated estimates of uncertainty (e.g. confidence intervals)
[ ]	[x]	For null hypothesis testing, the test statistic (e.g. *F*, *t*, *r*) with confidence intervals, effect sizes, degrees of freedom and *P* value noted<br>*Give P values as exact values whenever suitable.*
[ ]	[x]	For Bayesian analysis, information on the choice of priors and Markov chain Monte Carlo settings
[x]	[ ]	For hierarchical and complex designs, identification of the appropriate level for tests and full reporting of outcomes
x		Estimates of effect sizes (e.g. Cohen's *d*, Pearson's *r*), indicating how they were calculated

Our web collection on statistics for biologists contains articles on many of the points above.

Software and code

Policy information about availability of computer code

Data collection	Code present at: https://github.com/karlsmithbyrne/Pan__Cancer__Protein__MR__2024/blob/main/Manuscript__COde.
Data analysis	R version 4.2.1, tidyverse(2.0.0), ggplot2(3.4.4), TwoSampleMR package (0.5.6), Coloc package (5.2.3), plink (1.9)

For manuscripts utilizing custom algorithms or software that are central to the research but not yet described in published literature, software must be made available to editors and reviewers. We strongly encourage code deposition in a community repository (e.g. GitHub). See the Nature Portfolio guidelines for submitting code & software for further information.

Data

Policy information about availability of data

All manuscripts must include a data availability statement. This statement should provide the following information, where applicable:

- Accession codes, unique identifiers, or web links for publicly available datasets
- A description of any restrictions on data availability
- For clinical datasets or third party data, please ensure that the statement adheres to our policy

Summary statistics from Zheng et al. (2020) can be obtained from OpenGWAS (https://gwas.mrcieu.ac.uk/), from Folkersen et al (2020) at http://www.scallop-consortium.com, from Ferkingstad et al. (2021) at https://www.decode.com/summarydata/ , and from Pietzner et al. (2021) at https://omicscience.org. We obtained summary genetic association data on breast cancer risk from the Breast Cancer Association Consortium (https://bcac.ccge.medschl.cam.ac.uk/), ovarian cancer risk from the Ovarian Cancer Association Consortium (https://ocac.ccge.medschl.cam.ac.uk/), and endometrial cancer risk from the Endometrial Cancer Association Consortium (https://www.ebi.ac.uk/gwas/publications/30093612#study__panel). Approval was received to use restricted summary genetic association data from INTEGRAL ILCCO consortia after submitting a proposal to access this data. Summary genetic association data from these consortia can be accessed by contacting INTEGRAL ILCCO (rayjean.hung@lunenfeld.ca) (https://ilcco.iarc.fr). Approval was also received to use restricted summary genetic association data on pancreatic cancer risk via dbGaP release phs000206.v5.p3. To enquire about gaining access to summary genetic association data for renal and head and neck cancer risk, contact brennanp@iarc.fr. To enquire about gaining access to summary genetic association data for bladder cancer risk, contact bart.kiemeney@radboudumc.nl. Summary statistics for Malignant non-melanoma were obtained from OpenGWAS (https://gwas.mrcieu.ac.uk/).

Research involving human participants, their data, or biological material

Policy information about studies with human participants or human data. See also policy information about sex, gender (identity/presentation), and sexual orientation and race, ethnicity and racism.

Reporting on sex and gender	Some cancer endpoints, such as breast, ovary, and endometrial cancers, were conducted only on assigned female at birth participants. Otherwise, no additional sex-stratified results are present in this manuscript.
Reporting on race, ethnicity, or other socially relevant groupings	All analyses with the exception of some admixed participants in the GWAS of cancers of the head an neck were of European ancestry.
Population characteristics	No covariates were used in analyses conducted in this study
Recruitment	No participants were recruited in this study
Ethics oversight	As per the absence of any individual level data no ethics approval is reported

Note that full information on the approval of the study protocol must also be provided in the manuscript.

Field-specific reporting

Please select the one below that is the best fit for your research. If you are not sure, read the appropriate sections before making your selection.

[x] Life sciences [ ] Behavioural & social sciences [ ] Ecological, evolutionary & environmental sciences

For a reference copy of the document with all sections, see nature.com/documents/nr-reporting-summary-flat.pdf

Life sciences study design

All studies must disclose on these points even when the disclosure is negative.

Sample size	Sample size calculations were conducted using https://shiny.cnsgenomics.com/mRnd/ to inform the interpretation of findings generated in this study.
Data exclusions	We excluded genetic variants with low power indicated by an F statistic < 10 and clumped summary statistics at an r2 of 0.01 to derive independent variants.
Replication	We performed replication MR analyses where an external cancer GWAS was available in either Finngen or a meta-analysis of UKBB with Finngen. MR associations were observed for 29 of the 68 protein-cancer associations that we were able to investigate.
Randomization	Not applicable due to twosample mendelian randomisartion study design.
Blinding	Not applicable due to twosample mendelian randomisartion study design.

Reporting for specific materials, systems and methods

We require information from authors about some types of materials, experimental systems and methods used in many studies. Here, indicate whether each material, system or method listed is relevant to your study. If you are not sure if a list item applies to your research, read the appropriate section before selecting a response.

Materials & experimental systems

n/a	Involved in the study	
[x]	[ ]	Antibodies
[x]	[ ]	Eukaryotic cell lines
[x]	[ ]	Palaeontology and archaeology
[x]	[ ]	Animals and other organisms
[x]	[ ]	Clinical data
[x]	[ ]	Dual use research of concern
[x]	[ ]	Plants

Methods

n/a	Involved in the study	
[x]	[ ]	ChIP-seq
[x]	[ ]	Flow cytometry
[x]	[ ]	MRI-based neuroimaging
