# Supplementary material for: Identifying therapeutic targets for cancer among 2074 circulating proteins and risk of nine cancers
Source: Nat Commun. 2024 Apr 29;15:3621. doi: 10.1038/s41467-024-46834-3 (PMC11059161; doi:10.1038/s41467-024-46834-3)
Supplement: Supplementary file 4 — Description of Additional Supplementary Files [file 41467_2024_46834_MOESM4_ESM.docx]

**Description of Additional Supplementary Files**

**Supplementary Data 1:** Cis-pQTL used in single pQTL Wald ratio Mendelian randomisation analyses.

**Supplementary Data 2:** Results from cis-pQTL Wald ratio Mendelian randomisation analyses and colocalisation analyses for all cancer endpoints where Wald ratio Mendelian randomisation passes convetional significance. Mendelian randomisation analyses are two-sided and significance after multiple testing is indicated by 'Significance' column.

**Supplementary Data 3:** Results from Mendelian randomisation analyses and colocalisation analyses that passed correction for multiple testing as well as results from replication analyses for cancer risk associations where available.

**Supplementary Data 4:** Results from Mendelian randomisation analyses of cancer risk on protein levels for proteins that associate with risk of cancer after multiple testing and are supported by colocalisation analyses. All tests are two sided.

**Supplementary Data 5:** Results from cis-pQTL Wald ratio Mendelian randomisation analyses and colocalisation results for proteins with non-cancer endpoints for proteins identified to associate with cancer in main analyses after correction for multiple testing. Mendelian randomisation analyses are two-sided.

**Supplementary Data 6:** Results from hyprcolocalisation analyses with posterior probabilities for colocalisation between traits greater than 0.7 between proteins and cancer risk, and non-cancer endpoints identified to also be associated with cancer-risk.

**Supplementary Data 7:** Results for proteins identified as in either main analysis or drug target analyses with mapping to drug targets and highest level of therapeutic investigation
